# Imaging and Histopathologic Nuances of Epithelioid Glioblastoma

**DOI:** 10.1155/2018/1285729

**Published:** 2018-05-22

**Authors:** Brian H. Le, Richard A. Close

**Affiliations:** ^1^Department of Pathology, Reading Hospital, West Reading, PA 19611, USA; ^2^Neurosurgery-Tower Health Medical Group, West Reading, PA 19611, USA

## Abstract

A 27-year-old male without significant past medical history presented following collapse resulting from a syncopal episode at work. There was an episode of vomiting, and one of tonic-clonic seizure activity, which was spontaneously resolved after approximately one minute. His neurologic exam was nonfocal, with full strength in the bilateral upper and lower extremities, and no sensory deficits were elicited. MRI studies demonstrated a 4.7 cm rim-enhancing cystic mass in the right temporal-parietal region, with resultant mass effect and edema. At surgery, intraoperative pathologic consultation favoured a primary glial neoplasm. Subsequent complete histologic examination on permanent sections confirmed the presence of glioblastoma, with a morphologic pattern and immunohistochemical profile most consistent with epithelioid glioblastoma (WHO grade IV). Epithelioid glioblastoma is a rare, especially aggressive variant of IDH-wildtype glioblastoma, recognized in the 2016 World Health Organization classification. Approximately 50% of such tumors harbour the BRAF V600E mutation, which has also been observed in some melanomas where selective inhibitors have demonstrated a therapeutic role. The especially aggressive behaviour and poor clinical outcome typically observed for this variant of glioblastoma demonstrate the importance of emerging areas relevant to neurooncology, specifically those of proteomic characterization and therapeutic nanomedicine.

## 1. Introduction

The 2016 WHO Classification of Tumours of the Central Nervous System incorporates certain molecular data which serve as important prognostic and predictive markers into the diagnostic scheme for diffuse astrocytic and oligodendroglial tumors [1]. Most notably, isocitrate dehydrogenase (IDH) mutational status has been included to define diagnostic categories of astrocytomas. Based on the status of the IDH1 and IDH2 genes, glioblastoma, a grade IV tumor, is further stratified into IDH mutant, IDH wildtype, or not otherwise specified (NOS) if data pertaining to its IDH mutational status is incompletely elucidated. Among IDH-wildtype tumors, the WHO recognizes giant cell glioblastoma, gliosarcoma, and epithelioid glioblastoma [1]. In particular, the diagnosis of epithelioid glioblastoma carries a poor prognosis [1–3]. Interestingly, the BRAF V600E mutation is detected in about half of these tumors [1, 2, 4, 5]; although the possible therapeutic implications of BRAF inhibitors is not well studied.

## 2. Case Presentation

A 27-year-old male who had previously been in good health presented to the emergency room after he collapsed at work, with transient loss of consciousness. This was accompanied by subsequent vomiting. A neurologic examination was nonfocal, demonstrating full strength in the upper and lower extremities, without sensory deficits. However, the patient was amnestic to the events surrounding this syncopal episode and consequent collapse. A tonic-clonic seizure was observed, which spontaneously resolved after approximately one minute.

**Figure 1:**
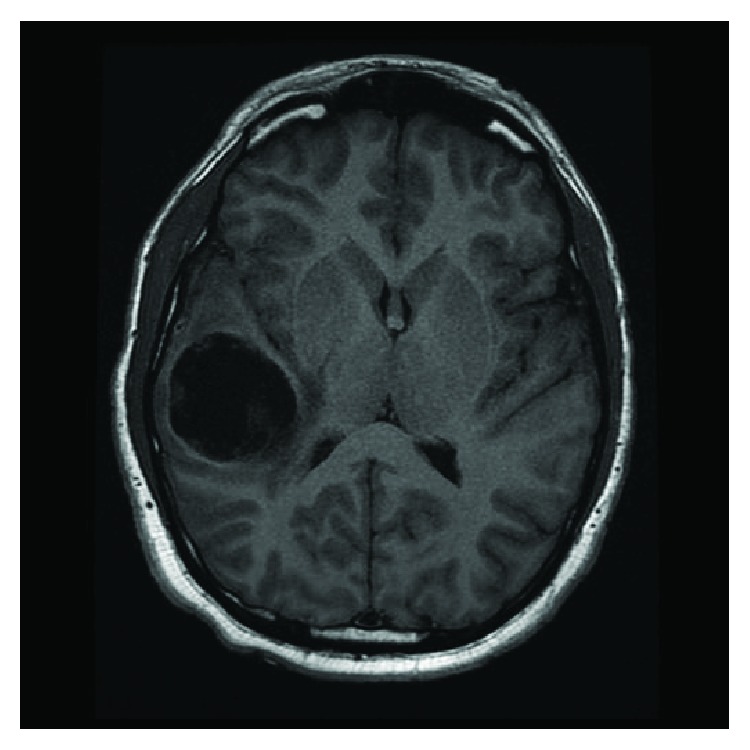
MRI showing a right temporal-parietal cystic mass that is T1 hypointense.

**Figure 2:**
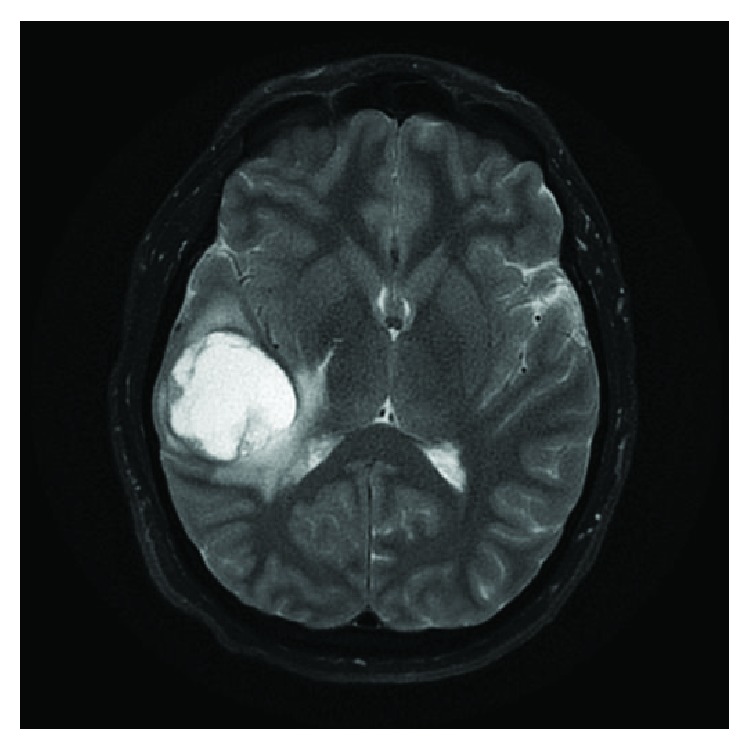
The cystic mass is hyperintense on T2-weighted MRI, with rim enhancement.

MRI studies demonstrated a 4.7 cm rim-enhancing cystic mass in the right temporal-parietal region, with resultant mass effects and edema, giving rise to an approximate 4 mm right to left midline shift. This mass was hypointense on T1 (Figure 1) and hyperintense on T2 (Figure 2). A lack of restricted diffusion argued against the differential diagnosis of abscess, thus favouring a cystic neoplasm. Subsequent CT scans of the chest, abdomen, and pelvis showed no mass lesions; as such, a primary central nervous system (CNS) neoplasm was favoured.

**Figure 3:**
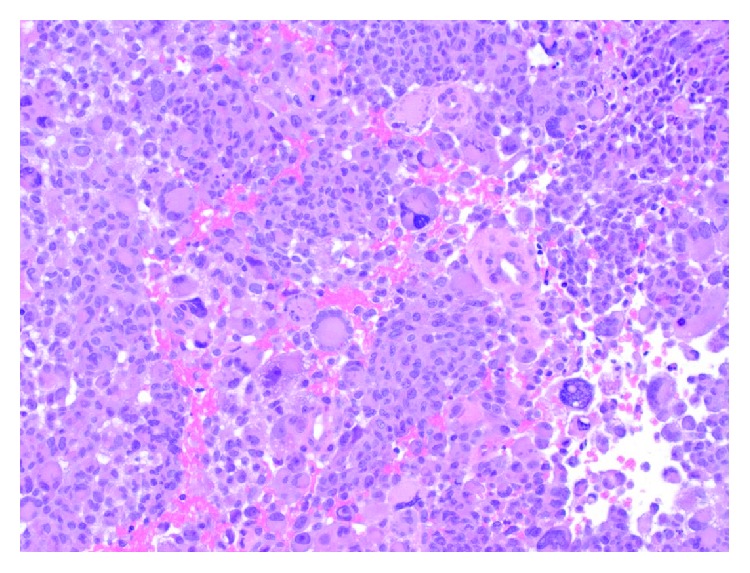
Intermediate power view of the tumor showing a cellular proliferation of large, epithelioid cells with abundant cytoplasm. Numerous multinucleated giants cells are present (H&E stain, 200x original magnification).

**Figure 4:**
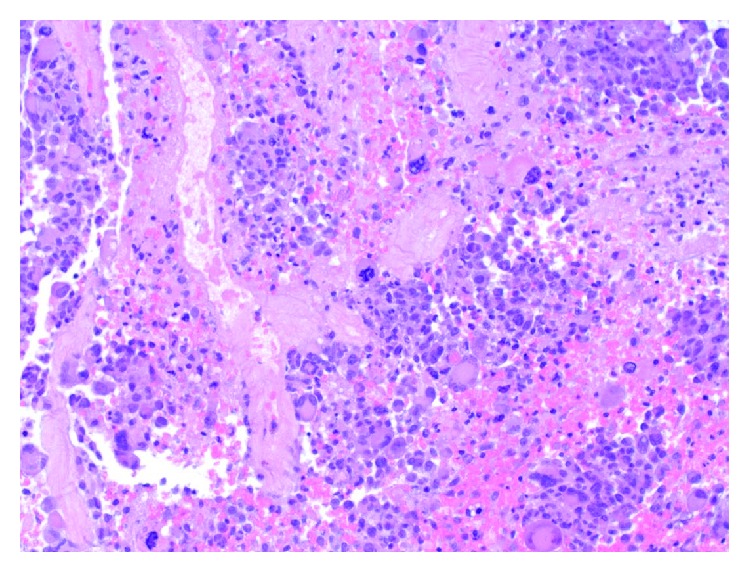
Significant variation in size and shapes (pleomorphism) is noted, with mitotic figures and regions of haemorrhage and necrosis (H&E stain, 400x original magnification).

**Figure 5:**
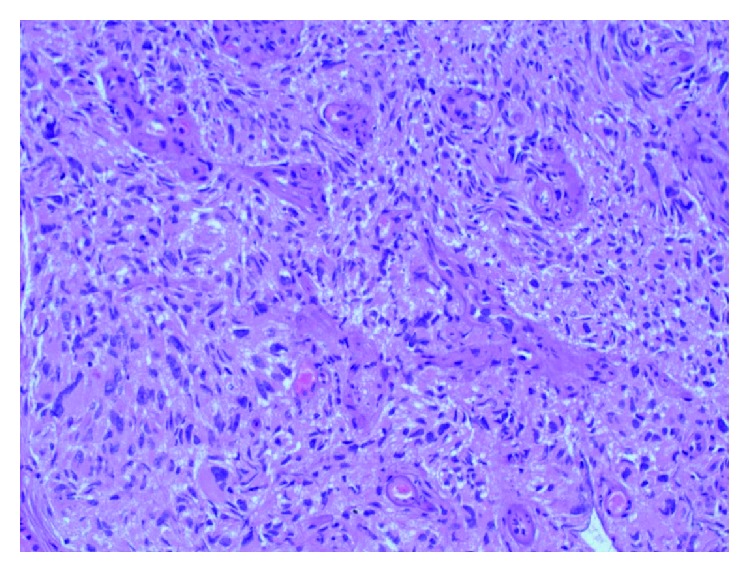
Microvascular proliferation is evident in some regions of the tumor (H&E stain, 200x original magnification).

**Figure 6:**
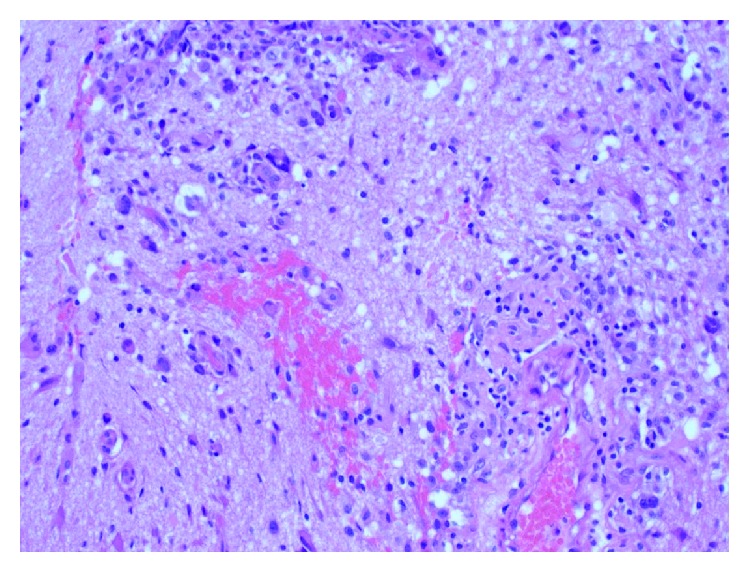
Intermediate power view of the tumor at interface with adjacent brain parenchyma, demonstrating an infiltrative border (H&E stain, 200x original magnification).

**Figure 7:**
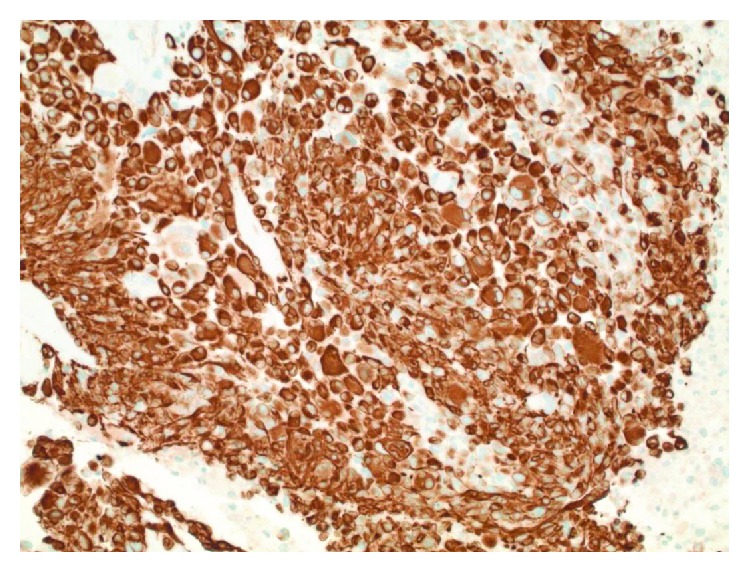
Diffuse immunoreactivity for GFAP confirms that the tumor is of glial-astrocytic origin (200x original magnification).

**Figure 8:**
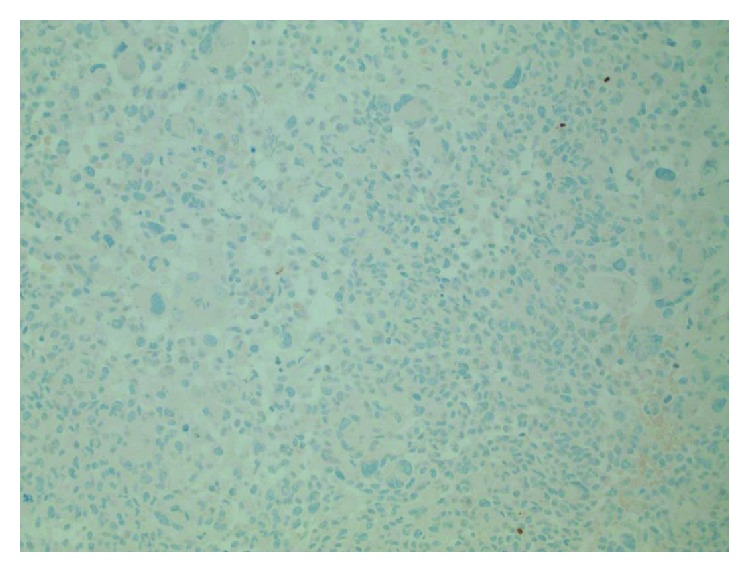
Immunohistochemistry for pancytokeratin shows no reactivity (positive control verified) (200x original magnification).

**Figure 9:**
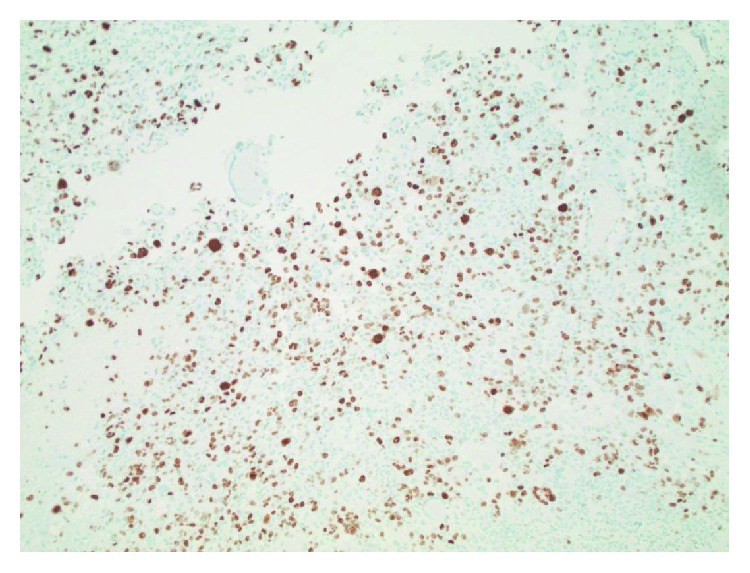
The Ki-67 proliferative index is markedly elevated, indicating a high-grade, aggressive lesion (100x original magnification).

At surgery, intraoperative pathologic consultation suggested a primary glial neoplasm. A maximal safe resection was performed. Permanent histologic sections show a cellular neoplasm composed of large, epithelioid cells, with numerous multinucleated giant cells (Figure 3). There is significant nuclear pleomorphism, with mitotic activity, haemorrhage, and necrosis (Figure 4). Microvascular proliferation is seen (Figure 5), and an infiltrative interface is observed with adjacent brain parenchyma (Figure 6). Neoplastic cells show diffuse reactivity for the glial fibrillary acidic protein (GFAP) (Figure 7) and S-100 protein, confirming glial origin. There is no reactivity for pancytokeratin or AE1/AE3 (Figure 8). Only faint, patchy reactivity is seen for synaptophysin, which accentuates predominantly background neuropil. The Ki-67 proliferative index is markedly elevated (Figure 9). There is no nuclear reactivity for p53 protein by immunohistochemistry, and no increase in reticulin deposition is noted with the reticulin stain. Subsequent molecular studies show no evidence of IDH1 or IDH2 mutations, and MGMT promoter methylation is not detected. However, the tumor demonstrates the BRAF V600E mutation. Globally considered, the findings are most in keeping with a diagnosis of epithelioid glioblastoma (WHO grade IV).

Following surgery, adjuvant radiation with concurrent temozolomide, followed by full dose temozolomide, were offered to the patient. He has also undergone rehabilitation therapy, and no adverse events have been reported at 3 months following surgery.

## 3. Discussion

The radiographic differential diagnosis for an enhancing intra-axial brain lesion is wide and include primary CNS neoplasms, metastatic tumors, demyelinating disease, and abscess. In this scenario, the patient's clinical symptoms, in the absence of a febrile illness and in correlation with MRI characteristics, favoured a primary neoplastic process. Although body-wide imaging was unrevealing of a probable primary site, solitary metastasis could not be entirely excluded on the basis that metastatic melanoma can present in any fashion.

Differential diagnoses for a cystic neoplasm in a young patient are typically those of a low grade neoplasm. However, the morphologic findings in this case, accompanied by an elevated Ki-67 proliferative index, indicated a high-grade lesion. As such, key histologic differential diagnostic considerations in this scenario shifted to metastasis, anaplastic xanthoastrocytoma (WHO grade III), giant cell glioblastoma (WHO grade IV), and epithelioid glioblastoma (WHO grade IV).

Expression of epithelial markers such cytokeratin and keratin AE1/AE3 can be problematic in distinguishing metastatic carcinoma from glial neoplasms, especially since epithelial and pseudoepithelial differentiation, along with expression of various cytokeratins, have been well established in glioblastomas [6]. Rodriguez and colleagues previously subclassified glioblastomas based on the nature of epithelioid morphology and keratin expression; in this scheme, adenoid glioblastoma (A-GBM) demonstrated architectural nests or cords and occasional pseudoglandular/cribriform spaces, without reactivity for low molecular weight keratins or polyclonal carcinoembryonic antigen (pCEA). Glioblastomas with true epithelial differentiation (TE-GBM) would be expected to show epithelial morphology architecturally, with squamous nests or true glandular structures, accompanied by immunoreactivity for epithelial markers such as cytokeratin. Epithelioid glioblastoma (E-GBM) would show large, round cells with abundant cytoplasm but would lack both glandular architecture and expression of markers associated with epithelial differentiation [6].

In the current case, diffuse reactivity for GFAP and S-100 protein, along with an elevated Ki-67 proliferative index and negative findings from body scans, indicated that this was a primary, high-grade astrocytoma. The absence of increased reticulin deposition argued against anaplastic xanthoastrocytoma and giant cell glioblastoma. Further support to exclude the possibility of giant cell glioblastoma resided in the observation that the tumor was nonreactive for p53 protein. The differential diagnosis of giant cell glioblastoma (IDH wildtype) is especially important to consider in this 27-year-old patient, as it is more commonly observed in younger individuals, typically circumscribed, and carries a somewhat better prognosis than most glioblastomas [1]. However, in this case while numerous giant cells were present in the tumor, they did not constitute the predominant cell type, and the expected diffuse nuclear reactivity for p53 was absent. Globally considered, a final diagnosis of epithelioid glioblastoma (WHO grade IV) was rendered and further supported by the presence of a BRAF V600E mutation, in the absence of IDH1 and IDH2 mutations. In consideration of the classification scheme elaborated by Rodriguez and colleagues, the absence of immunoreactivity for two epithelial markers (pancytokeratin and AE1/AE3A) also suggested pseudoepithelial differentiation as opposed to true epithelial differentiation; as such, this lesion would also be classified as an epithelioid glioblastoma, or E-GBM, under such scheme.

Epithelioid glioblastoma, as a rare variant of IDH-wildtype glioblastoma, is especially aggressive [1, 3, 5]. They are more frequently encountered in younger patients, show early recurrence, and demonstrate rapid progression and leptomeningeal infiltration [3, 5]. As targeted inhibitory antibody therapy has recently gained increasing attention in oncology, point mutation of the BRAF gene at codon 600 (BRAF V600E) may present as a target for potential treatment [2, 7]. Interestingly, approximately half of epithelioid glioblastomas harbour the BRAF V600E mutation, thus exhibiting morphologic and molecular overlap with pleomorphic xanthoastrocytoma [1–5]. Brown and colleagues have reported successful treatment of two patients with BRAF V600E mutant pleomorphic xanthoastrocytoma using the BRAF inhibitor dabrafenib in combination with trametinib [7]. Recent studies suggest that routine screening for BRAF V600E mutations should be considered for glioblastomas in patients below the age of 30, particularly those that demonstrate epithelioid features [2].

The aggressive behaviour and overall poor prognosis for glioblastoma, especially for this rare variant in a young patient, invite exploration of proteomic characterization. The study of proteomics in tumors aims to characterize proteins, along with their function, in the intracellular and extracellular spaces [8]. Although many molecular genetic assays are now part of the standard classification scheme for glioblastoma, as reflected in the WHO guidelines [1], DNA and RNA levels reflect duplication/replication and transcription and do not always correlate with protein abundance at the translational level, where the major proteomic constituents of glioblastoma are not fully known [9]. Song and colleagues recently performed proteomic profiling of gliomas, identifying 36 molecules that were commonly changed at the level of the gene and protein, 200 proteins with high mutation rates among glioblastomas, and 14 genes with high-level protein modification [9]. Candidate proteins and pathways that may give rise to temozolomide resistance have also been studied [10]. Large-scale genomic and proteomic analysis efforts in glioblastoma have also uncovered potential drug targets [10, 11].

Furthermore, the poor prognosis associated with glioblastoma despite adjuvant chemotherapy and radiation therapy calls for future consideration of therapeutic nanomedicine. Although radiation therapy has been demonstrated to improve prognosis in glioblastoma, the true benefit of chemotherapy may be deemed as controversial, as the blood-brain barrier poses a challenge to drug delivery towards tumor targets [12]. After all, capillary endothelial cells form a tight regulatory boundary between the blood stream and brain parenchyma. Nanomedicine offers a potential strategy to overcome this challenge by providing alternate vehicles for drug delivery that may optimize passage through the blood-brain barrier. Nanoparticles, defined as any particle of any shape and material below 1 micron in diameter, have shown enhanced permeation and retention (EPR), being able to pass through vasculature and be retained in tumor target cells, without returning freely to the circulation. Studies have demonstrated the ability of nanoconjugates to effectively cross the blood-brain barrier, concentrating in tumor cells [12, 13]. A wide variety of biocompatible nanoparticles are being researched, showing promise for improved, targeted delivery of not only chemotherapeutic agents, but also agents that aid in imaging [12].
